# Dieting and Food Cue-Related Working Memory Performance

**DOI:** 10.3389/fpsyg.2016.01944

**Published:** 2016-12-14

**Authors:** Adrian Meule

**Affiliations:** ^1^Department of Psychology, University of SalzburgSalzburg, Austria; ^2^Center for Cognitive Neuroscience, University of SalzburgSalzburg, Austria

**Keywords:** diet, dieting success, restrained eating, working memory, executive functioning, food cues

## Abstract

Executive functioning (e.g., working memory) is tightly intertwined with self-regulation. For example, food cue-elicited craving has been found to impair working memory performance. Furthermore, current dieters have been found to show lower working memory performance than non-dieters. Recent research, however, suggests that it is crucial to consider dieting success in addition to current dieting status or restrained eating in order to reveal cognitive mechanisms that are associated with successful eating-related self-regulation. The current study investigated food cue-related working memory performance as a function of dieting status and dieting success in female students. Participants performed an *n*-back task with pictures of food and neutral objects. Reaction time in response to food pictures was slower than in response to neutral pictures, whereas omission errors did not differ between picture types. Current food craving was increased after performing the food block, but not after the neutral block. There was an indirect effect of current dieting status on higher food craving after the food block, which was mediated by slower reaction time to food vs. neutral pictures. Furthermore, higher dieting success was associated with fewer omission errors in the food vs. neutral block in current dieters. There were no relationships of restrained eating with current food craving and task performance. Results further highlight the need to differentiate between successful and unsuccessful dieting in addition to current dieting status or restrained eating when examining possible mechanisms of overeating or successful restraint. Although palatable food cues induce food craving regardless of dieting success, they may boost executive functioning in successful dieters, which helps them to overcome these temptations.

## Introduction

Restrained eating refers to the intention to restrict food intake deliberately in order to prevent weight gain or to promote weight loss (Tuschl, 1990). Higher scores on restrained eating (particularly when assessed with the *Restraint Scale*, RS) are associated with a tendency to overeat and higher body mass index (BMI; Stroebe, 2008). This has led to the proposition that dieting (i.e., caloric restriction) may increase the incentive salience of palatable foods (e.g., Fedoroff et al., 2003), which may in turn threaten dieting success. Restraint scores alone, however, do not provide information about if a person is currently dieting or not and, if a person is currently dieting, if that person is successful or unsuccessful in restricting food intake (Lowe, 1995).

Increasing evidence suggests that incorporating perceived self-regulatory success in dieting appears to be an important predictor of cognitive processing of and responses to palatable food cues. For example, self-perceived dieting success has been found to be differentially related to activation and inhibition of dieting goals in response to palatable food cues (Papies et al., 2008; Stroebe et al., 2008; Van Koningsbruggen et al., 2011a,b, 2012), to facets of food craving experiences (Meule et al., 2012a), to the use of dietary control strategies (Meule et al., 2011), and to food intake in the laboratory (Houben et al., 2012; Friese et al., 2015). Differential associations have also been reported with general measures of self-regulatory ability such as impulsivity or cardiac autonomic regulation (Meule et al., 2012b; van Koningsbruggen et al., 2013). Moreover, successful dieters exhibited better executive functioning as measured by motor response inhibition in response to high-calorie food cues (Houben et al., 2012; Meule et al., 2014a).

Response inhibition refers to controlling one’s behavior to override a strong internal predisposition or external lure (Diamond, 2013) and, thus, its relevance for dietary self-control is apparent (Hall, 2016). However, other domains of executive functioning have been identified as crucial for self-regulatory processes as well (Hofmann et al., 2012). Working memory, for example, refers to holding information in mind and mentally working with it (Diamond, 2013). Therefore, working memory and inhibition support one another and rarely is one needed but not the other (Diamond, 2013). High working memory capacity has been suggested to contribute to more effective self-regulation in several self-regulatory domains (Hofmann et al., 2011). Considerable evidence suggests that experiencing craving (e.g., for food), consumes cognitive resources and, thus, impairs performance on tasks that require working memory (Kemps et al., 2008; Tiggemann et al., 2010; May et al., 2012). Moreover, it has been found that introducing an interfering working memory load reduces food craving (Kemps and Tiggemann, 2010, 2015).

With regard to dieting behavior, it has been found that current dieters display lower working memory performance than non-dieters and this impairment is partly related to preoccupying thoughts about food, weight, and shape (Kemps and Tiggemann, 2005; Kemps et al., 2005). These studies, however, assessed general working memory performance, but did not measure working memory performance related to relevant cues (i.e., food). Moreover, it was not differentiated if dieters were successful or unsuccessful. In a recent study, it could be shown that successful dieters’ reaction times were less affected by food cues in a working memory task, indicating that dieting success may indeed moderate food cue-related working memory performance (Higgs et al., 2015).

In the current study, food cue-related working memory performance was investigated as a function of both current dieting status and dieting success with a version of the *n*-back task. Based on a previous study (Meule et al., 2012d), it was expected that working memory performance would be reduced (i.e., longer reaction times, higher number of omission errors) in response to food cues as compared to neutral cues. Furthermore, it was expected that dieters would demonstrate reduced working memory performance compared to non-dieters (Kemps and Tiggemann, 2005; Kemps et al., 2005). This reduced performance was expected to be observed particularly in response to food cues in unsuccessful dieters, but not in successful dieters (who may even show better task performance in response to food cues, similar to what has been found in motor response inhibition tasks; Houben et al., 2012; Meule et al., 2014a). Finally, it was explored if lower working memory performance was associated with higher subsequent food craving and, therefore, if task performance mediated a possible association between dieting status and/or dieting success and food cue-induced craving.

## Materials and Methods

### Participants

This study adhered to the guidelines outlined in the Declaration of Helsinki as revised in 2008. Seventy female students (*M*_age_ = 22.0 years, *SD* = 3.28; *M*_BMI_ = 21.5 kg/m^2^, *SD* = 2.82) participated in exchange for course credits. Except for gender, no inclusion or exclusion criteria were applied. Mean food deprivation (i.e., time since last meal) was *M* = 4.57 h (*SD* = 5.11). Twenty-four participants (34.3%) indicated that they were currently dieting.

### Measures and Materials

#### Dieting Status

Dieting status (yes/no) was assessed with a single question (“Are you currently restricting your food intake to control your weight [e.g., by eating less or avoiding certain foods]?”; cf. Meule et al., 2012b).

#### Perceived Self-Regulatory Success in Dieting (PSRS)

Dieting success was assessed with the PSRS (Fishbach et al., 2003; Meule et al., 2012c). This three-item questionnaire asks participants how successful they are in watching their weight and losing extra weight, and how difficult it is for them to stay in shape. Responses are scored on a seven-point scale (1–7), anchored *not successful/not difficult* and *very successful/very difficult*. After reverse coding the third item, all items are summed up and, thus, scores can ranged between three and 21. Higher scores indicate higher perceived self-regulatory success. Internal consistency was α = 0.633 in the current study. Note that the term *dieting success* when referring to PSRS scores will be used throughout this manuscript for the sake of brevity, although other descriptions such as *successful weight regulation* would also be appropriate (Houben et al., 2012; Friese et al., 2015).

#### Restraint Scale (RS)

Restrained eating was assessed with the RS (Herman et al., 1978; Dinkel et al., 2005). This ten-item questionnaire asks participants about their general concern for dieting and weight fluctuations. Responses are scored on a four-point (0–3, five items) and five-point (0–4, five items) scale, with response categories ranging, for example, from *never/not at all* to *always/extremely*. All items are summed up and, thus, scores can range between zero and 35. Higher scores indicate stronger restrained eating tendencies. Internal consistency was α = 0.770 in the current study.

#### Food Cravings Questionnaire-State (FCQ-S)

Current food craving was assessed with the FCQ-S (Cepeda-Benito et al., 2000; Meule et al., 2012a). This 15-item questionnaire asks participants about the intensity of their momentary craving for specific foods. Responses are scored on a five-point scale (1–5), with response categories ranging from *strongly disagree* to *strongly agree*. All items are summed up and, thus, scores can range between 15 and 75. Higher scores indicate more intense current food craving. Internal consistencies ranged from α = 0.872 to α = 0.919 in the current study.

#### n-back Task

**FIGURE 1:**
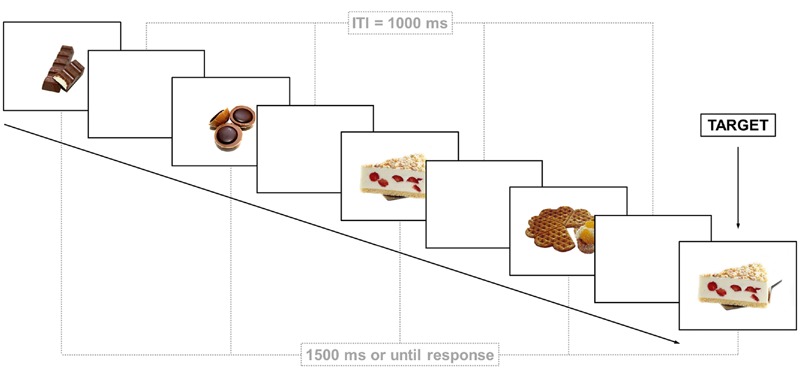
**Representative screen displays of trials in the food block of the *n*-back task.** Participants were instructed to press a key in response to pictures that have been displayed two trials earlier. ITI = Inter-trial interval.

Thirty pictures of high-calorie foods (both savory and sweet foods) and 30 pictures of neutral stimuli (flowers, office supplies, household items) were selected from the *food.pics* database^1^ (Blechert et al., 2014).^2^ Food pictures did not contain meat or fish because vegetarians were not excluded from the study. Food and neutral pictures did not differ in jpg file size, visual complexity, and contrast [all *t*s_(58)_ < 1.48, *p*s > 0.140]. All foods displayed on the food images were high caloric (*M* = 354.77 kcal/100g, *SD* = 148.01; *M* = 736.95 kcal/image, *SD* = 832.70). The *n*-back task was compiled with E-prime 2.0 (Psychology Software Tools Inc., Pittsburgh, PA, USA) and displayed on an LCD TFT 22″ monitor. In this task, stimuli are presented one-by-one and subjects are instructed to press a button whenever a stimulus is presented that is the same as the one presented *n* trials previously (so-called *targets*; in this case, it was a 2-back task; **Figure 1**). Participants first performed a practice block with numbers, which consisted of 14 trials, and received feedback in case of a false response. The test phase consisted of a block with food pictures and a block with neutral pictures (order of blocks was counterbalanced across subjects). Each picture was presented four times, but only once as a target, resulting in 120 trials in each block including 30 targets. Order of trials was pseudo-randomized such that order of target trials was equal in both blocks. Each picture was presented for 1500 ms or until a response was made. Between trials, a blank screen was displayed for 1000 ms (**Figure 1**). Thus, each block had a duration of approximately 5 min.

### Procedure

Participants were instructed to refrain from eating at least 1 h before the experiment to ensure that they were not fully sated. Participants were tested individually in the laboratory. After arrival, they read and signed informed consent and completed the FCQ-S for the first time. Then, they performed the practice block and the first test block. Participants then completed the FCQ-S for the second time, before performing the second test block. After the task, they completed the FCQ-S for the third time. At the end of the experiment, participants completed the PSRS, RS, and other questionnaires. Finally, height and weight were measured.

### Data Analyses

Trials with a reaction time of ≤150 ms were excluded from analyses (cf. Meule et al., 2012d). Measures of interest in the *n*-back task were reaction time (*M* = 584 ms, *SD* = 100) and number of omission errors (*M* = 9.67 errors, *SD* = 6.41). Commission errors (i.e., pressing the button in response to non-targets) were rare (*M* = 4.09 errors, *SD* = 2.89) and not further analyzed.

Associations between participant characteristics (age, BMI, food deprivation) and questionnaire measures (dieting status, dieting success, restrained eating, current food craving) were examined with *t*-tests and correlations (**Table 1**). Multilevel models were calculated with HLM version 7.01 (Raudenbush et al., 2011) to examine associations between task performance and questionnaire measures. Specifically, block type (food block = 1, neutral block = 2) was entered at level 1 and dieting status (1 = dieting, 2 = non-dieting), dieting success, and an interaction between dieting status × dieting success were entered at level 2 for predicting reaction time (**Table 2**) and omission errors (**Table 3**). Similar models were calculated using restrained eating instead of dieting and dieting success as predictor variable (**Tables 4** and **5**). As there was a large variation in food deprivation (i.e., time since last meal; see Participants section), it was included as a control variable at level 2 in all models. Significant interactions were followed up with *t*-tests and linear regression analyses, as described below.

**Table 1:** Descriptive statistics for food deprivation, age, body mass index (BMI), dieting success, and restrained eating in the full sample and as a function of dieting status and correlations between variables.

	Full sample (*n* = 70)			Dieters (*n* = 24)			Non-dieters (*n* = 46)			*t*-test statistics		*r*				
	*M*	*SD*	Range	*M*	*SD*	Range	*M*	*SD*	Range	*t*	*p*	1.	2.	3.	4.	5.
1. Food deprivation (hours)	4.57	5.11	1–26	5.44	6.44	1–26	4.12	4.26	1–17	0.90	0.372	–	-0.011	0.075	0.105	0.292^∗^
2. Age (years)	22.0	3.28	18–40	22.1	2.58	19–30	21.9	3.61	18–40	0.23	0.819		–	0.068	0.036	-0.067
3. Body mass index (kg/m^2^)	21.5	2.82	15.4–34.1	22.4	3.66	17.8–34.1	21.0	2.16	15.4–26.7	1.70	0.100			–	-0.432^∗^	0.466^∗^
4. Perceived Self-Regulatory Success in Dieting Scale	12.4	3.20	6–19	11.5	3.60	6–16	12.8	2.91	6–19	1.72	0.090				–	-0.418^∗^
5. Restraint Scale	12.1	4.92	2–27	15.5	4.76	8–27	10.4	4.06	2–24	4.71	<0.001					–

*^∗^*p* < 0.05.*

**Table 2:** Coefficients with robust standard errors of the mixed model with dieting status and dieting success as predictors of reaction time.

Effect	Coefficient	*SE*	*p*
Intercept (γ_00_)	678	65.2	<0.001
Level 1			
Block type (γ_10_)	-103	37.9	0.008
Level 2			
Food deprivation (γ_01_)	-0.36	3.15	0.908
Dieting status (γ_02_)	-38.8	35.8	0.282
Dieting success (γ_03_)	-38.4	62.5	0.541
Dieting status × dieting success (γ_04_)	38.7	36.9	0.298
Cross-level interactions			
Food deprivation (γ_11_)	-0.31	1.88	0.869
Dieting status (γ_12_)	47.7	20.3	0.022
Dieting success (γ_13_)	-49.0	32.2	0.132
Dieting status × dieting success (γ_14_)	21.7	17.7	0.224

*Level 1 model equation: reaction time_ij_ = β_0j_ + β_1j_ × block type_ij_ + *r*_ij_. Level 2 model equation: β_0j_ = γ_00_ + γ_01_ × food deprivation_j_ + γ_02_ × dieting status_j_ + γ_03_ × dieting success_j_ + γ_04_ × (dieting status × dieting success)_j_ + *u*_0j_, β_1j_ = γ_10_ + γ_11_ × food deprivation_j_ + γ_12_ × dieting status_j_ + γ_13_ × dieting success_j_ + γ_14_ × (dieting status × dieting success)_j_ + *u*_1j_. Block type was coded as 1 = food block, 2 = neutral block. Dieting status was coded as 1 = dieting, 2 = non-dieting. Food deprivation was grand mean-centered. Dieting success was *z*-standardized before calculating the product term.*

**Table 3:** Coefficients with robust standard errors of the mixed model with dieting status and dieting success as predictors of omission errors.

Effect	Coefficient	*SE*	*P*
Intercept (γ_00_)	4.79	1.88	0.013
Level 1			
Block type (γ_10_)	0.40	1.23	0.744
Level 2			
Food deprivation (γ_01_)	-0.06	0.10	0.538
Dieting status (γ_02_)	0.03	1.28	0.983
Dieting success (γ_03_)	-7.28	1.62	<0.001
Dieting status × dieting success (γ_04_)	4.67	1.20	<0.001
Cross-level interactions			
Food deprivation (γ_11_)	0.04	0.08	0.612
Dieting status (γ_12_)	-0.29	0.80	0.716
Dieting success (γ_13_)	3.72	1.21	0.003
Dieting status × dieting success (γ_14_)	-2.31	0.79	0.005

*Level 1 model equation: omission errors_ij_ = β_0j_ + β_1j_ × block type_ij_ + *r*_ij_. Level 2 model equation: β_0j_ = γ_00_ + γ_01_ × food deprivation_j_ + γ_02_ × dieting status_j_ + γ_03_ × dieting success_j_ + γ_04_ × (dieting status × dieting success)_j_ + *u*_0j_, β_1j_ = γ_10_ + γ_11_ × food deprivation_j_ + γ_12_ × dieting status_j_ + γ_13_ × dieting success_j_ + γ_14_ × (dieting status × dieting success)_j_ + *u*_1j_. Block type was coded as 1 = food block, 2 = neutral block. Dieting status was coded as 1 = dieting, 2 = non-dieting. Food deprivation was grand mean-centered. Dieting success was *z*-standardized before calculating the product term.*

**Table 4:** Coefficients with robust standard errors of the mixed model with restrained eating as predictor of reaction time.

Effect	Coefficient	*SE*	*p*
Intercept (γ_00_)	618	16.9	<0.001
Level 1			
Block type (γ_10_)	-22.1	8.91	0.016
Level 2			
Food deprivation (γ_01_)	-0.35	3.41	0.918
Restrained eating (γ_02_)	1.69	5.31	0.752
Cross-level interactions			
Food deprivation (γ_11_)	-0.57	2.44	0.818
Restrained eating (γ_12_)	-2.97	2.01	0.144

*Level 1 model equation: reaction time_ij_ = β_0j_ + β_1j_ × block type_ij_ + *r*_ij_. Level 2 model equation: β_0j_ = γ_00_ + γ_01_ × food deprivation_j_ + γ_02_ × restrained eating_j_ + *u*_0j_, β_1j_ = γ_10_ + γ_11_ × food deprivation_j_ + γ_12_ × restrained eating_j_ + *u*_1j_. Block type was coded as 1 = food block, 2 = neutral block. Dieting status was coded as 1 = dieting, 2 = non-dieting. Food deprivation and restrained eating were grand mean-centered.*

**Table 5:** Coefficients with robust standard errors of the mixed model with restrained eating as predictor of omission errors.

Effect	Coefficient	*SE*	*p*
Intercept (γ_00_)	5.29	0.79	<0.001
Level 1			
Block type (γ_10_)	-0.30	0.45	0.507
Level 2			
Food deprivation (γ_01_)	-0.10	0.12	0.434
Restrained eating (γ_02_)	-0.05	0.16	0.776
Cross-level interactions			
Food deprivation (γ_11_)	0.05	0.08	0.525
Restrained eating (γ_12_)	0.06	0.09	0.486

*Level 1 model equation: omission errors_ij_ = β_0j_ + β_1j_ × block type_ij_ + *r*_ij_. Level 2 model equation: β_0j_ = γ_00_ + γ_01_ food deprivation_j_ + γ_02_ × restrained eating_j_ + *u*_0j_, β_1j_ = γ_10_ + γ_11_ × food deprivation_j_ + γ_12_ × restrained eating_j_ + *u*_1j_. Block type was coded as 1 = food block, 2 = neutral block. Dieting status was coded as 1 = dieting, 2 = non-dieting. Food deprivation and restrained eating were grand mean-centered.*

Mediation analyses were conducted with PROCESS for SPSS (Hayes, 2013) to examine an indirect effect of dieting behavior on food craving after each block via task performance (reaction time, omission errors). Food craving before the *n*-back task was included as covariate. Indirect (i.e., mediation) effects were evaluated with bias-corrected bootstrap confidence intervals based on 10,000 bootstrap samples.

## Results

### Associations between Participant Characteristics and Questionnaire Measures

Current dieters did not differ from non-dieters in age, BMI, dieting success, and food deprivation, but had higher scores on restrained eating (**Table 1**). Dieting success was uncorrelated with age and food deprivation, but negatively correlated with restrained eating and BMI. Restrained eating was uncorrelated with age, but positively correlated with BMI and food deprivation (**Table 1**).

Food craving was higher after the food block (*M* = 39.3, *SD* = 11.5) than before the task [*M* = 34.9, *SD* = 9.40, *t*_(68)_ = 5.92, *p* < 0.001] and compared to food craving after the neutral block [*M* = 34.5, *SD* = 10.3, *t*_(67)_ = 7.13, *p* < 0.001]. Food craving before the task and after the neutral block did not differ from each other [*t*_(68)_ = 0.37, *p* = 0.716]. Current dieters did not differ from non-dieters in current food craving before the task, after the food block, and after the neutral block (all *t*s < 0.77, *p*s > 0.448). Dieting success and restrained eating were uncorrelated with current food craving before the task, after the food block, and after the neutral block (all *r*s < 0.165, *p*s > 0.177).

### Task Performance

Block type significantly predicted reaction time (**Table 2**). As the food block was coded with 1 and the neutral block was coded with 2, the negative coefficient indicates that reaction time was slower in the food block (*M* = 595 ms, *SD* = 103) than in the neutral block (*M* = 573 ms, *SD* = 112). Dieting status showed a cross-level interaction with block type when predicting reaction time (**Table 2**). To follow up the nature of this interaction, paired *t*-tests were calculated, comparing reaction time in the food and neutral block in dieters and non-dieters separately. In current dieters, reaction time in the food block (*M* = 590 ms, *SD* = 104) was slower than in the neutral block [*M* = 543 ms, *SD* = 122, *t*_(23)_ = 2.39, *p* = 0.03]. In non-dieters, reaction time did not differ between block types (*t*_(45)_ = 0.98, *p* = 0.332).

**FIGURE 2:**
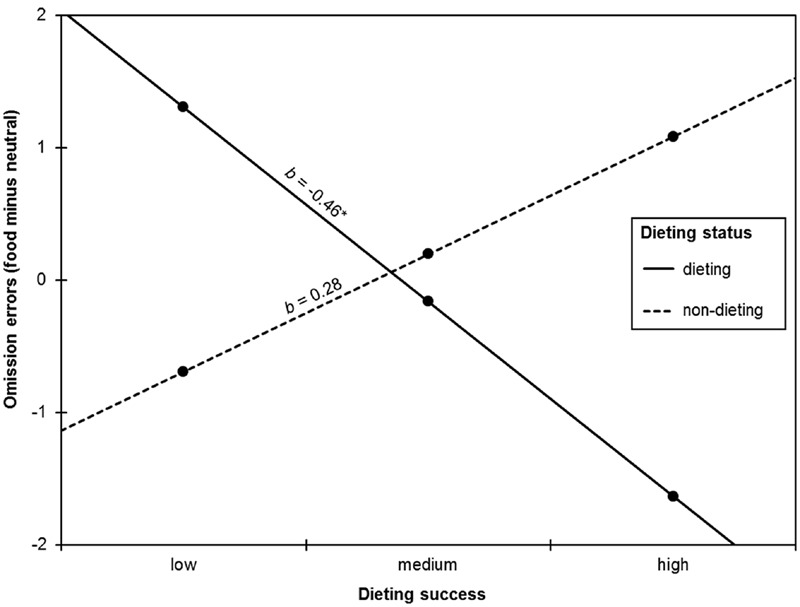
**Simple slopes probing the interaction of dieting status and dieting success when predicting omission errors in the food block minus omission errors in the neutral block.** There was a negative association between dieting success and omission errors in current dieters, but not in non-dieters. The asterisk indicates *p* < 0.05.

Dieting success significantly predicted omission errors on level 2 and in cross-level interaction with block type (**Table 3**). These effects, however, were further qualified by a dieting status × dieting success cross-level interaction with block type (**Table 3**). To follow up the nature of this interaction, a regression analysis was calculated with dieting status, dieting success (mean centered), and the interaction dieting status × dieting success predicting an omission errors difference score (i.e., omission errors in the food block minus omission errors in the neutral block). Dieting status (*b* = 0.36, *SE* = 0.96, *p* = 0.710) and dieting success (*b* = 0.03, *SE* = 0.15, *p* = 0.864) did not predict omission errors difference score. However, there was a significant interaction between dieting status × dieting success (*b* = 0.74, *SE* = 0.29, *p* = 0.013). Probing this interaction revealed that dieting success was negatively associated with the number of omission errors in current dieters, but not in non-dieters (**Figure 2**). Specifically, current dieters made more omission errors in the food relative to the neutral block with decreasing dieting success or, vice versa, current dieters made fewer omission errors in the food relative to the neutral block with increasing dieting success.

Restrained eating did not predict task performance (**Tables 4** and **5**). The only significant predictor of task performance in these models was block type when predicting reaction time (**Table 4**), similar to the model described above (**Table 2**).

### Mediation Analyses

**FIGURE 3:**
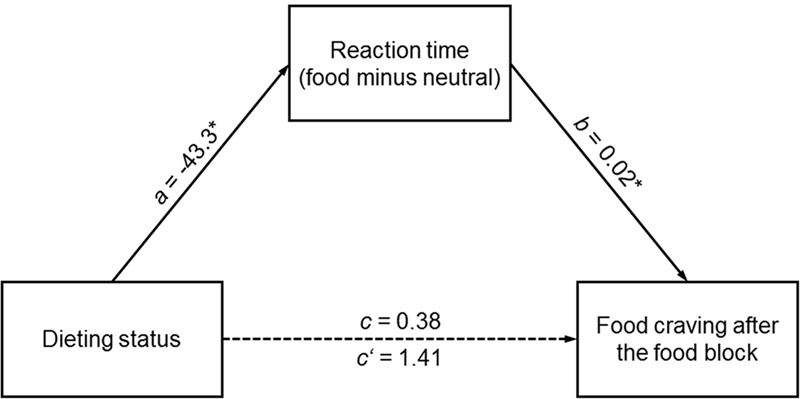
**Mediation model of an indirect effect of dieting status (independent variable) on food craving after the food block (outcome variable) via reaction time in the food block minus reaction time in the neutral block (mediator).** Food craving before the task was entered as covariate. The dashed line indicates that there was an indirect effect of dieting status on food craving after the food block (bootstrap estimate -1.03, 95%CI [-3.19, -0.03]) in the absence of a total (*c*) or direct (*c’*) effect. Asterisks indicate *p* ≤ 0.05.

There was an indirect effect of dieting status on food craving after the food block via reaction time difference score (i.e., reaction time in the food block minus reaction time in the neutral block; **Figure 3**). That is, being a dieter was indirectly related to higher food craving after the food block compared to being a non-dieter. This effect, however, was not directly observable, but mediated by current dieters’ slower reactions in the food block relative to reaction time in the neutral block. A similar analysis with food craving after the neutral block as outcome variable did not indicate such an indirect effect (bootstrap estimate -0.41, 95%CI [-1.82, 0.15]). Furthermore, similar mediation models with omission errors difference score as mediator, which additionally included dieting success and the interaction of dieting status × dieting success as predictors (moderated mediation, model no. 7 in PROCESS) did not reveal any indirect effects.

## Discussion

In the current study, female students performed a working memory task with food and neutral stimuli. Food craving was higher after the food block compared to before and compared to food craving after the neutral block, indicating that the palatable food cues used in the *n*-back task induced craving. Reactions in response to food cues were slower than in response to neutral cues in general and in current dieters in particular. Mediation analyses showed that there was an indirect effect of dieting status on current food craving after the food block via reaction time. Specifically, current dieters reacted slower in response to food cues relative to neutral cues and these reactions were, in turn, predictive of higher subsequent food craving. In contrast to previous studies (Kemps and Tiggemann, 2005; Kemps et al., 2005), current dieters did not show impairments in working memory performance in general. This might be explained by methodological differences between studies as Kemps et al. (2005) employed working memory span tasks, which have been argued to require more subcomponents of executive functions (Diamond, 2013).

While the finding of slower reaction time to food cues replicates findings from a previous study with a similar *n*-back task (Meule et al., 2012d), these slowed reactions were predictive of more intense food craving, which was not found in the previous study. One reason for this discrepancy might be that current food craving was only assessed once after completion of the entire task in the previous study (Meule et al., 2012d), but was not measured promptly after each block, like it was done in the present study. Nevertheless, results are in line with studies in which induction of food craving resulted in a slowing of responses in subsequent reaction time tasks (e.g., Kemps et al., 2008) and with a study showing that slowed responses in response to food cues in a motor inhibition task predicted subsequent food craving (Meule et al., 2014b). Furthermore, the current study is the first to show that current dieters are more prone to this slowing of reactions to food cues compared to non-dieters, which mediates experiencing more intense food craving subsequently.

In contrast to a previous finding (Meule et al., 2012d), omission errors did not differ between the food and the neutral block, which may be related to changes in stimulus selection and design (e.g., no differentiation between sweet and savory foods). However, it was found that dieting status and dieting success interactively predicted the number of omission errors: higher dieting success was associated with fewer omission errors in response to food cues relative to neutral cues in current dieters. Of note, this effect was not associated with current food craving, that is, successful dieters experienced just as much food craving as unsuccessful dieters did. This is in line with other studies reporting that exposure to palatable food cues increases food craving to an equal extent in successful and unsuccessful dieters (Houben et al., 2012), at least when they are hungry (Meule et al., 2014a). Although successful and unsuccessful dieters appear to be equally tempted by palatable food, successful dieters seem to be better able to resist these temptations. This is also in line with the goal conflict model of eating behavior, which posits that “even though palatable food stimuli also prime the eating enjoyment goal [in successful dieters], the increased accessibility of the dieting goal helps them to inhibit eating enjoyment and to engage in healthy eating” (Stroebe et al., 2013, p. 125). One mechanism of this successful self-regulation appears to be exertion of inhibitory control over appetitive responses toward palatable food as has been demonstrated by better motor response inhibition in food-related behavioral tasks (Houben et al., 2012; Meule et al., 2014a). The current results extend these findings to another domain of executive functioning, namely working memory. Similar to results from behavioral inhibition tasks (Houben et al., 2012; Meule et al., 2014a), successful dieters did not show better task performance in general, but specifically in response to food cues. Thus, it appears that palatable food cues boost executive functioning in successful dieters, leading to better working memory performance and inhibitory control (or, vice versa, that food cues impair executive functioning in unsuccessful dieters).

Restrained eating was unrelated to current food craving and task performance in the current study. Similarly, other researchers have argued that previous experiences with successful weight control (i.e., PSRS scores) are a more important predictor of disinhibition to food cues (or disinhibited food intake; Houben et al., 2012). The current results extend these observations by highlighting the importance of further differentiating between current dieting and non-dieting in addition to successful and unsuccessful weight regulation. This may be necessary as it appears that there is a subgroup of individuals with high PSRS scores who are not concerned with regulating eating and weight (Nguyen and Polivy, 2014). Although restrained eating (as measured with the RS in particular) has been consistently found to be associated with overeating and higher BMI (Stroebe, 2008), it appears that taking current dieting status and dieting success into account may be better suited for identifying mechanisms that predict successful or unsuccessful eating- and weight regulation (Stroebe et al., 2013).

Interpretation of results is limited to young women and, thus, future studies are necessary that extend findings to other samples such as men or samples with a higher range in age and BMI. Furthermore, laboratory food intake was not measured in the current study and it would be worthwhile to examine if increased food craving or a higher number of omission errors in response to food cues does actually translate to increased food consumption after the task. Finally, while dieting status, dieting success, and food craving were measured with self-report questionnaires in the current study, future studies may investigate if task performance in such an *n*-back task will be able to predict actual dieting behavior and food intake in daily life, for example, as assessed with ecological momentary assessment.

Given the findings obtained in the current study, future research may examine if food-related working memory performance can be improved by repeated training and if this translates into higher dieting success. Although working memory trainings have been found to increase working memory performance, it appears that these effects do not generalize to measures of “real-world” cognitive skills (Schwaighofer et al., 2015; Melby-Lervåg et al., 2016). Recent studies suggest, however, that general working memory trainings may indeed modify eating-related outcomes. In a study with obese children, for example, a combined inhibition and working memory training attenuated weight regain after an inpatient weight-loss treatment, albeit this effect was no longer present after 12 weeks (Verbeken et al., 2013). Most recently, a working memory training has been found to reduce eating concerns and emotional eating in obese adults (Houben et al., 2016). Similar to studies, in which food-related motor response inhibition trainings are used in an effort to modify eating behavior (Stice et al., 2016), future studies may develop and examine the effectiveness of food-related working memory trainings. Possibly, such trainings may facilitate dieting success by strengthening executive functions while at the same time reducing food cue reactivity through food cue exposure (Jansen et al., 2015).

## Author Contributions

AM conceived the study, analyzed the data, and wrote the manuscript.

## Conflict of Interest Statement

The author declares that the research was conducted in the absence of any commercial or financial relationships that could be construed as a potential conflict of interest.

## Footnotes

1 www.food-pics.sbg.ac.at

2 Picture number in the *food.pics* database: food items – 6, 9, 10, 22, 27, 38, 41, 42, 56, 71, 82, 83, 89, 106, 111, 113, 115, 116, 120, 133, 137, 142, 143, 145, 159, 165, 166, 167, 172, 173; neutral items – 1000, 1001, 1002, 1003, 1004, 1005, 1007, 1009, 1011, 1012, 1013, 1014, 1015, 1016, 1019, 1020, 1021, 1023, 1024, 1025, 1026, 1028, 1029, 1030, 1031, 1032, 1033, 1034, 1035, 1036.
